# Comparative Effectiveness of Switching From First-Generation Basal Insulins to Either Glargine 300 U/mL or Degludec 100 U/mL in Children and Adolescents With Type 1 Diabetes: Results From the ISPED CARD Clinical Registry

**DOI:** 10.1155/pedi/5514402

**Published:** 2025-11-21

**Authors:** Ivana Rabbone, Riccardo Bonfanti, Giusi Graziano, Fortunato Lombardo, Antonio Nicolucci, Marco Marigliano, Maria Chiara Rossi, Giacomo Vespasiani, Valentino Cherubini

**Affiliations:** ^1^Division of Pediatrics, Department of Health Sciences, Università del Piemonte Orientale, Novara, Italy; ^2^Department of Pediatrics, Pediatric Diabetology Unit, Diabetes Research Institute, IRCCS Ospedale San Raffaele, Milan, Italy; ^3^CORESEARCH – Center for Outcomes Research and Clinical Epidemiology, Pescara, Italy; ^4^Department of Human Pathology in Adult and Developmental Age “Gaetano Barresi”, University of Messina, Messina, Italy; ^5^Department of Surgery, Dentistry, Pediatrics and Gynecology, Section of Pediatric Diabetes and Metabolism, University of Verona, Verona, Italy; ^6^Meteda Srl, San Benedetto del Tronto (AP), Italy; ^7^Department of Women's and Children's Health, Azienda Ospedaliero-Universitaria, Ospedali Riuniti di Ancona, G. Salesi Hospital, Ancona, Italy

**Keywords:** basal insulin analogues, clinical registry, degludec 100 U/mL, glargine 300 U/mL, pediatric diabetes, real-world evidence, type 1 diabetes

## Abstract

**Background:**

To assess the real-world effectiveness of switching from first-generation basal insulins (1BIs) to either glargine U300 (Gla-300) or degludec U100 (Deg-100) in children and adolescents with type 1 diabetes (T1D), using data from the Italian ISPED CARD clinical registry.

**Materials and Methods:**

This multicenter retrospective observational study included 1063 pediatric patients with T1D from 22 diabetes centers across Italy who switched from 1BI to either Gla-300 (64.6%) or Deg-100 (35.4%) between 2021 and 2023. Propensity score matching (PSM) was applied to create comparable groups (*n* = 353 per group). Primary endpoint was the change in HbA1c at 6 months. Secondary endpoints included fasting blood glucose (FBG), standardized body mass index (BMI/SDS), and insulin doses at 6 and 12 months. Longitudinal models for repeated measures were used to assess treatment effectiveness.

**Results:**

Both groups showed significant and clinically relevant reductions in HbA1c at 6 months from ~ 8.7% to ~ 7.4% (−1.3 percentage points), maintained at 12 months, with no significant differences between groups. FBG also decreased significantly in both groups, slightly favoring Deg-100, but without statistical significance between groups. BMI/SDS remained stable. Gla-300 was associated with a slight increase in basal insulin dose over 12 months, while Deg-100 showed a temporary reduction at 6 months. A significant reduction in short-acting insulin dose (−0.03 U/kg) was observed in both groups.

**Conclusion:**

Switching from 1BI to either Gla-300 or Deg-100 significantly improves glycemic control in pediatric T1D patients without weight gain. Although both insulins showed comparable effectiveness, differences in titration patterns highlight the need for individualized treatment strategies and improved clinician education in insulin optimization. Safety outcomes, particularly hypoglycemia, could not be assessed.

## 1. Introduction

Technological progress has significantly transformed the management of type 1 diabetes (T1D) in pediatric patients. In particular, the development of insulin pumps, advancements in continuous glucose systems, and a wider use of the hybrid closed loop have markedly improved diabetes care in children and adolescents. However, it should be noted that the choice between pump therapy and multiple daily injections should be individualized, considering the child's age, lifestyle, ability to manage the device, and family support. Furthermore, young people may refuse or discontinue therapy using an insulin pump. Discontinuation is higher in adolescents and young adults, and the most frequently reported reasons include problems with wearability, technological difficulties, greater sense of disease, difficulties in doing sports, embarrassment, adhesions and pain in the place of needle insertion, disliking the pump or feeling anxious, and problems with glycemic control [1–4].

Therefore, the treatment with basal-bolus insulin schemes still plays an important role. In this respect, we have recently been facing significant advancements in insulin therapy, particularly concerning basal insulin formulations. Over recent years, newer second-generation basal insulins (2BIs), particularly insulin glargine U300 (Gla-300) and insulin degludec U100 (Deg-100) have been developed to improve glycemic control, reduce hypoglycemia risk, offer more flexible dosing options. These basal insulins offer longer durations of action and reduced variability profiles compared to first-generation basal insulin (1BI) analogs [5, 6]. These properties are supposed to contribute to improved glycemic control, potentially leading to lower HbA1c levels in children and adolescents. Gla-300 is indicated for the treatment of adolescents and children aged ≥ 6 years, while Deg-100 is approved for use in children as young as 1 year old.

Randomized clinical trials in adult [7] and pediatric [8, 9] T1D populations have demonstrated the advantages of 2BIs over 1BIs, thanks to their physiological profiles, dosing flexibility, and safety. These attributes are considered crucial for achieving optimal glycemic control, reducing acute and chronic complications, and enhancing the quality of life (QoL) for individuals with T1D [10]. Notably, the InRange study, utilizing clinically relevant continuous glucose monitoring (CGM) metrics, reported that Gla-300 is non-inferior to Deg-100 in adults with T1D, with comparable hypoglycemia and safety profiles [11]. Similarly, the real-world RESTORE-1 study, a retrospective chart review of over 1000 Italian adults with T1D, found that switching from 1BIs to either Gla-300 or Deg-100 resulted in similar improvements in glycemic control without significant weight gain [12]. These findings highlight the potential benefits of 2BIs in optimizing T1D management across different age groups.

In Italy, the ISPED CARD initiative, launched in 2019 [13], aligns with other large-scale data analysis projects such as the Italian AMD Annals [14, 15], the European DPV and SWEET projects [16, 17], and the U.S. T1D Exchange project [18]. The ISPED CARD database, which is periodically updated with retrospective data from electronic medical records (EMRs) across a network of pediatric diabetes clinics, facilitates the assessment and improvement of care quality and the evaluation of treatment effectiveness and long-term outcomes.

A previous analysis of the ISPED CARD database, involving 200 Italian T1D children and adolescents, demonstrated substantial HbA1c reductions following the switch from 1BI to Gla-300, sustained over 12 months [19]. Building on this, the present study aims to compare the real-world effectiveness of switching from 1BI to either Gla-300 or Deg-100 in a larger cohort of children and adolescents within the Italian pediatric usual care setting. By leveraging the comprehensive data within the ISPED CARD registry, we seek to provide valuable insights into the comparative effectiveness and safety of these insulin transitions in this T1D pediatric population.

## 2. Materials and Methods

ISPED CARD is an observational, retrospective, multicenter study, based on registry data anonymously extracted from EMRs. The study involved a network of 22 diabetes centers (among 59 registered Italian pediatric diabetes clinics) located in different areas of Italy [20].

All participating centers adopted the same EMR (MetaClinic - METEDA SRL, San Benedetto del Tronto (AP), Italy), allowing the extraction of a standard, reproducible set of data. Centers and patients were anonymous, and data were extracted and transferred via a standardized and validated secure procedure.

The following inclusion criteria were applied: male or female, aged from 0 to 18 years, diagnosis of T1D, switching to Gla-300 or Deg-100 from 1BI (i.e., regular human, glargine-100, detemir, or NPH). Exclusion criteria were prescription of another basal insulin analogue after initiating Gla-300 or Deg-100 and available follow-up shorter than 3 months.

The following characteristics were considered to describe the baseline patient profile: age, gender, diabetes duration, HbA1c, fasting blood glucose (FBG), standardized body mass index (BMI/SDS), lipid profile, blood pressure, and insulin therapy (available in EMRs as anatomical therapeutic chemical [ATC] codes).

Endpoints considered included the changes in HbA1c at 6 months (T6) (primary endpoint) and 12 months (T12) and the changes in FBG, BMI/SDS, and insulin doses at T6 and T12.

The study protocol was approved by all local ethics committees of the participating centers.

### 2.1. Statistical Analysis

Characteristics of patients who switched to Gla-300 or Deg-100 in the years 2021–2023 were compared using Mann–Whitney *U* test for continuous variables and chi-square test for categorical variables. *p*-values <0.05 were statistically significant.

Propensity score (PS) matching was applied to ensure the balance of the following case-mix variables: age, sex, duration of diabetes, HbA1c, FBG, BMI/SDS, and per-kg dose of basal and rapid insulin. Standardized mean difference lower than 0.10 (absolute value) indicated a good balance.

Longitudinal models for repeated measures were applied to evaluate the comparative effectiveness of Gla-300 vs. Deg-100 on HbA1c, FBG, and BMI/SDS. The titration of basal and rapid insulins over time was also evaluated.

The following evaluation times were considered

• T0: first prescription of Gla-300 or Deg-100 in the years 2021–2023 (index date, baseline).
• T6: 6-month follow-up (data recorded in EMR after 1–6 months from the index date).
• T12: 1-year follow-up (data recorded in EMR after 7–12 months from the index date).

HbA1c effectiveness at 6 months represented the primary endpoint.

The results are expressed as estimated means at each follow-up and as estimated mean changes compared to T0 and related 95% confidence intervals (95% CI). The “within-group *p*-values” resulting from the models indicate whether, for each endpoint, the changes in the mean values estimated at each follow-up within each group were statistically significant compared to the baseline. The values of the “between-group *p*-values” resulting from the models indicate whether the mean changes in the parameters at each follow-up were significantly different between the two groups. *p*-values <0.05 were considered as statistically significant.

Analyses were performed using SAS software, version 9.4 (SAS Institute Inc., North Carolina, USA).

## 3. Results

In the years 2021–2023, a total of 1063 T1D patients routinely attending 22 pediatric diabetes centers changed their therapy by switching from 1BI to 2BI, of whom 687 (64.6%) were prescribed Gla-300 and 376 (35.4%) Deg-100.

Characteristics of these patients are shown in Table 1, which provides information on the phenotype of patients switching from 1BI to Gla-300 or Deg-100. Patients switching to Gla-300 were significantly older than patients switching to Deg-100 (13.1 vs. 11.7 years; *p* < 0.0001), while there were no systematic differences between the two groups regarding sex, duration of diabetes, and FBG and BMI/SDS levels at baseline. As regards type of previous basal and short-acting insulin, the differences were marginal. In fact, almost all patients were previously treated with Glargine 100 U/mL (99.7% in the Gla-300 group and 100% in the Deg-100 group) and Lispro or Aspart short-acting insulin (89.1% in Gla-300 group and 93.4% in Deg-100 group). Insulin doses were comparable between the two groups.

The two groups were PS matched by age, representing the only unbalanced characteristic. After PS matching, 353 patients per group were identified. Post-matching balance of the following case-mix variables was further verified: age, sex, duration of diabetes, HbA1c, FBG, BMI/SDS,and per-kg dose of basal and short-acting insulin. After applying PS matching, all the selected case-mix variables were well balanced (SMD less than 0.10; Table 2).

**Figure 1:**
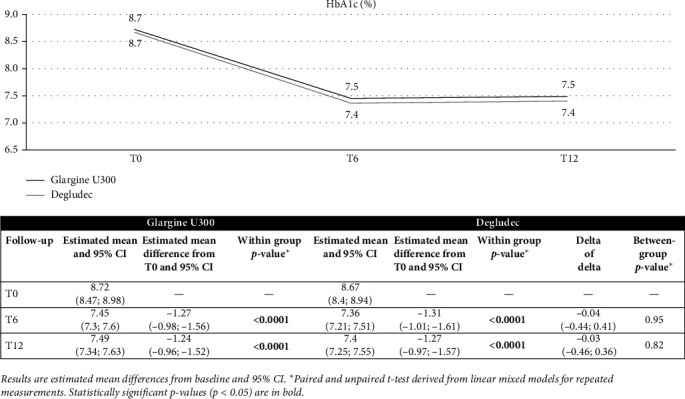
Changes from T0 to T12 in estimated mean HbA1c levels.

Figure 1 and Table 3 show the results of the longitudinal models for repeated measures relative to temporal trends of HbA1c, FBG, BMI/SDS, and insulin doses over 12 months.

The analysis shows a statistically significant and clinically relevant reduction in mean HbA1c values of 1.3% after 6 months in both groups (primary endpoint). The reduction obtained was maintained at 12 months without statistically significant difference between the two groups. Notably, HbA1c fell more in children <12 years than in adolescents (12–18 years), regardless of the 2BI used. In children, at 12 months HbA1c decreased by −1.96% (95% CI −1.49 to −2.43) with Gla-300 and −1.73% (95% CI −1.23 to −2.23) with Deg-100 (between-group *p*=0.94). In adolescents, the reductions were −0.72% (95% CI −0.39 to −1.05) with Gla-300 and −0.93% (95% CI −0.58 to −1.28) with Deg-100 (between-group *p*=0.29).

Furthermore, the analysis showed a statistically significant and clinically relevant reduction at T6 in mean FBG values with both 2BIs. The reduction obtained was maintained at 12 months in both groups.

No change in the mean BMI/SDS values in both groups emerged.

In terms of insulin doses, data show an increase in the per-kg dose of Gla-300, which was statistically significant at T12. Conversely, there was a significant reduction at T6 in the per-kg dose of Deg-100. The per-kg dosage was overall higher with Gla-300 both at 6 months (*p*=0.003) and at 12 months (*p*=0.01).

In both groups, there was a significant reduction of 0.03 U/Kg of short-acting insulin dose at 6 months.

## 4. Discussion

This large-scale, real-world analysis demonstrates the comparative effectiveness of switching from 1BIs to either Gla-300 or Deg-100 in a cohort of 1063 children and adolescents with T1D in Italy. A notable difference in prescription patterns was observed, with a higher proportion of patients switching to Gla-300 compared to Deg-100. This is likely influenced by local prescribing practices and clinical preferences. Crucially, the distinct age-related prescription limitations of these insulins also played a role. Deg-100, approved for use in children as young as 1 year old, was more frequently prescribed in younger patients. Conversely, Gla-300, approved for adolescents and children aged ≥6 years, was prescribed to an older cohort, resulting in a significant age difference between the groups at baseline. To mitigate the potential bias introduced by this age difference, a critical confounder in pediatric diabetes management, propensity score matching (PSM) was employed. Both insulin analogs yielded significant and clinically relevant reductions in HbA1c (−1.3% at 6 months) and FBG, without inducing weight gain, underscoring their efficacy in improving glycemic control in this population in real life. Benefits were larger in younger children, who mostly rely on parents regarding diabetes management vs. adolescents who are reaching adulthood. However, in both age groups reductions in HbA1c levels were substantial. If sustained over the long term, these reductions in HbA1c levels could have a significant impact on the development of complications and on QoL, facilitating the achievement of recommended therapeutic targets.

These findings are consistent with previous studies in adult populations, which have shown the benefits of 2BIs in terms of improved glycemic control and reduced variability [12]. The observed HbA1c reductions align with those reported in both randomized clinical trials and real-world studies [21]. However, the pediatric population presents unique challenges, including the influence of growth, puberty, insulin sensitivity, and therapy adherence (young children supervised by parents vs. teenagers with growing therapy autonomy) on dosing and management strategies, and this study provides valuable real-world evidence of the effectiveness of these insulin transitions in children and adolescents.

Distinct dosing patterns were observed in this study: the per-kg dose of Gla-300 increased over 12 months, while the Deg-100 dose decreased at 6 months before returning to baseline at 12 months. This suggests potential differences in dosing requirements and titration strategies between the two insulins. Specifically, it is well-known that a higher increase in the dose of Gla-300 vs Gla-100 could be needed to optimize his effect and potentiate its clinical benefit (as per Summary of Product Characteristics). However, the observed differences in basal insulin dosing patterns may reflect the need for individualized titration strategies, considering the dynamic physiological changes during growth and puberty.

The reduction in short-acting insulin requirements observed with both 2BIs is likely related to their more stable and prolonged pharmacodynamic profiles, resulting in a flatter and more predictable basal coverage. This allows a better balance between basal and bolus components and reduces the need for prandial corrections.

The significant improvements in glycemic control observed in this study emphasize the importance of timely switching from 1BI to 2BI in pediatric T1D. However, despite the well-documented safety profiles of 2BIs, suboptimal titration practices were evident. This highlights the need for clinicians to adopt a more proactive approach to insulin titration to achieve optimal glycemic targets. The persistent issue of suboptimal titration, also noted in the EDITION JUNIOR trial [8], suggests a potential knowledge gap or a lack of confidence in managing these newer insulin analogs. This is also due to the fact that, unlike in adults, no standardized titration algorithms are typically applied in children. ISPAD guidelines recommend individual adjustment under medical supervision rather than automatic titration rules [22]. Insulin dose adjustment—both basal and bolus—is therefore highly individualized and frequently modified by the diabetes care team and progressively by families and patients themselves. Children present a high variability in insulin sensitivity (related to growth, puberty, physical activity, and meals), which makes fixed titration schedules less safe. However, enhanced education and training for healthcare providers are crucial to maximize the benefits of 2BIs in pediatric T1D. Moreover, the study underscores the importance of individualized titration, structured education, and adherence strategies.

This study boasts several strengths, including its large sample size, which allows for robust comparative analyses of Gla-300 and Deg-100 in a real-world setting. The use of propensity score matching minimized potential confounding effects, ensuring a balanced comparison between the two groups. However, the study is not without limitations. The retrospective nature of the analysis precludes the assessment of safety outcomes, as detailed data on hypoglycemia and other adverse events were not consistently available in the EMRs. The lack of this information may stem from the tendency to record hypoglycemia data in free-text fields, making it unusable for statistical purposes. Moreover, it is likely that in many cases glycemic profiles are analyzed using each device's specific platform without downloading the data into the EMR. For the same reason, CGM metrics were seldom available. Time in range, time below range, and glycemic variability data would have strengthened the paper and position it within the current landscape of diabetes technology. Future studies should aim to incorporate comprehensive safety data, including information from CGM and patient-reported outcomes.

Also, the reasons for the switch were unknown and could differ between groups, thus representing a possible unknown confounder. However, the very high HbA1c levels at the time of the switch in both groups suggest that unsatisfactory metabolic control may represent the most frequent reason for changing therapy.

Finally, results derive from an Italian cohort of children and adolescents attending pediatric diabetes centers. It remains to be established whether our findings are generalizable to other populations or healthcare settings, considering differences in healthcare systems or prescribing practices.

## 5. Conclusion

In conclusion, this large-scale, real-world study demonstrates that switching from 1 BI to either Gla-300 or Deg-100 results in comparable and significant improvements in glycemic control, without weight gain, in children and adolescents with T1D. These findings support the use of 2BIs as effective alternatives to 1BIs in this population. However, the observed suboptimal insulin titration highlights the need for enhanced clinician education and more aggressive titration strategies to achieve optimal glycemic targets. Future research should focus on evaluating the long-term safety and efficacy of these insulin transitions, incorporating detailed safety data and patient-reported outcomes. The ISPED CARD initiative, with its ongoing efforts to collect comprehensive safety data, will play a crucial role in advancing our understanding of these therapies in the Italian pediatric T1D population.

## Figures and Tables

**Table 1:** Characteristics of the population in patients switching from 1BI to 2BI in the years 2021–2023 before PS matching.

Variable	*N*	Switch to glargine U300	*N*	Switch to degludec	*p*-Value^a^	SMD^a^
*N*		687		376		
T1D duration (years) (mean ± sd)	387	4.8 ± 3.3	180	5.0 ± 3.5	0.41	0.07
Gender (%)	687		376			0.04
Women		46.0		47.9	0.56	
Men		54.0		52.1		
Age (years) (mean ± sd)	687	13.1 ± 3.2	376	11.7 ± 4.2	<0.0001	0.36
Basal insulin dose/die (U/Kg)	616	0.4 ± 0.2	280	0.4 ± 0.1	0.97	0.003
Short-acting insulin dose/die (U/Kg)	481	0.5 ± 1.1	207	0.5 ± 0.3	0.42	0.08
HbA1c (%) (mmol/mol)	604	8.5 ± 2.1	290	8.7 ± 2.3	0.35	0.07
Fasting blood glucose (mg/dl) (mmol/l)	174	223.1 ± 143.9	102	229.2 ± 143.4	0.73	0.04
BMI/SDS (mean ± sd)	402	1.0 ± 1.0	192	1.1 ± 0.8	0.39	0.08
BMI/SDS >1.5 (%)	400	25.5	191	28.8	0.40	0.07
LDL > 100 mg/dl (%)	259	35.9	101	33.7	0.69	0.05
Blood pressure >140/70 mmHg (%)	518	18.1	221	19.9	0.57	0.04

*Note:* Data are mean and standard deviation or proportion.

Abbreviation: SMD, standardized mean difference.

^a^ *p*-Values derived from Mann–Whitney *U* test for continuous variables and chi-square test for categorical variables.

**Table 2:** Characteristics of the population in patients with switch from 1BI to 2BI in the years 2021–2023 after PS matching (matching variables: age).

Variable	*N*	Switch to glargine U300	*N*	Switch to degludec	*p*-Value^a^	SMD^a^
*N*	—	353	—	353	—	—
T1D duration (years) (mean ± sd)	187	4.9 ± 3.5	179	5.0 ± 3.5	0.75	0.03
Gender (%)	353	—	353	—	0.88	0.01
Women	—	46.7	—	47.3	—	—
Men	—	53.3	—	52.7	—	—
Age (years) (mean ± sd)	353	12.4 ± 3.5	353	12.2 ± 3.7	0.60	0.04
Previous basal insulin	353	—	353	—	0.56	0.11
	—	2 (0.7)	—	0 (0.0)	—	—
Basal insulin dose/die (U/Kg)	318	0.4 ± 0.2	265	0.4 ± 0.1	0.61	0.04
Short-acting insulin dose/die (U/Kg)	245	0.5 ± 0.2	203	0.5 ± 0.3	0.64	0.04
HbA1c (%) (mmol/mol)	307	8.7 ± 2.2	272	8.7 ± 2.3	0.78	0.02
Fasting blood glucose (mg/dl) (mmol/l)	94	227.7 ± 150.8	97	228.3 ± 139.7	0.98	0.004
BMI/SDS (mean ± sd)	206	1.1 ± 1.2	182	1.1 ± 0.8	0.87	0.02
BMI/SDS >1.5 (%)	206	27.2	181	29.3	0.65	0.05
LDL > 100 mg/dL (%)	141	36.2	92	30.4	0.37	0.12
Blood pressure >140/70 mmHg (%)	258	17.8	215	20.0	0.55	0.05

*Note:* Data are mean and standard deviation or proportion.

Abbreviation: SMD, standardized mean difference.

^a^ *p*-Values derived from Mann–Whitney *U* test for continuous variables and chi-square test for categorical variables.

**Table 3:** Changes from T0 to T12 in estimated mean in secondary outcomes.

Endpoints	Visit	Glargine U300			Degludec				
		Estimated mean and 95% CI	Estimated mean difference and 95% CI	*p*-Value	Estimated mean and 95% CI	Estimated mean difference and 95% CI	Within group *p*-value*⁣*^*∗*^	Delta of delta	Between-group *p*-value*⁣*^*∗*^
FBG (mg/dl)	T0	228 (199; 257)	—	—	230 (202; 259)	—	—	—	—
	T6	179 (159; 199)	−48.9 (−12.0;−85.9)	**0.009**	148 (131; 165)	−82.4 (−47.9;−116.9)	**<** **0** **.0001**	−33.5 (−82.2; 19.9)	0.23
	T12	167 (152; 183)	−60.8 (−28.8;−92.7)	**0.0002**	140 (125; 155)	−90.2 (−58.7;−121.6)	**<** **0** **.0001**	−29.4 (−72.4; 18.5)	0.24

BMI/SDS	T0	1.08 (0.94; 1.21)			1.00 (0.86; 1.14)				
	T6	1.00 (0.89; 1.11)	−0.08 (0.05;−0.2)	0.23	0.99 (0.88; 1.09)	−0.01 (0.12;−0.14)	0.85	0.07 (−0.11; 0.27)	0.41
	T12	1.09 (0.98; 1.21)	0.02 (0.15;−0.11)	0.79	1.00 (0.89; 1.12)	0.01 (0.15;−0.13)	0.93	−0.01 (−0.21; 0.1)	0.48

Basal insulin (U/Kg)	T0	0.36 (0.35; 0.38)	—	—	0.37 (0.35; 0.39)	—	—	—	
	T6	0.37 (0.36; 0.39)	0.01 (0.03;0)	0.18	0.34 (0.32; 0.36)	−0.03 (−0.01;−0.05)	**0.0007**	−0.04 (−0.06;−0.01)	**0.003**
	T12	0.39 (0.37; 0.41)	0.03 (0.05; 0.01)	**0.0003**	0.37 (0.35; 0.39)	0.00 (0.02;−0.02)	0.81	−0.03 (−0.06;−0.01)	**0.01**

Short-acting insulin (U/Kg)	T0	0.46 (0.43; 0.49)	—	—	0.45 (0.42; 0.49)	—	—	—	
	T6	0.43 (0.4; 0.46)	−0.03 (−0.01;−0.06)	**0.02**	0.42 (0.39; 0.45)	−0.03 (0;−0.06)	**0.04**	0.00 (−0.04; 0.05)	0.73
	T12	0.44 (0.41; 0.48)	−0.02 (0.01;−0.05)	0.17	0.43 (0.4; 0.47)	−0.02 (0.01;−0.05)	0.22	0.00 (−0.04; 0.05)	0.79

*Note:* Results are estimated mean differences from baseline and 95% CI.

*⁣* ^*∗*^Paired and unpaired *t*-test derived from linear mixed models for repeated measurements. Statistically significant *p*-values (*p* < 0.05) are in bold.

## Data Availability

The datasets generated during and/or analyzed during the current study are available from the corresponding author upon reasonable request. Qualified researchers may request access to patient-level data and related documents (including, e.g., the clinical study report, study protocol with any amendments, blank case report form, statistical analysis plan, and dataset specifications). Patient-level data will be anonymized, and study documents will be redacted to protect the privacy of trial participants.
